# Predictive Value of [^18^F]FDG PET/CT for Neoadjuvant Chemoradiotherapy Response in Nasopharyngeal Carcinoma

**DOI:** 10.3390/jcm14186508

**Published:** 2025-09-16

**Authors:** Natale Quartuccio, Federico Sireci, Sabina Pulizzi, Stefania Nicolosi, Dante D’Oppido, Salvatore Ialuna

**Affiliations:** 1 Nuclear Medicine Unit, Ospedali Riuniti Villa Sofia-Cervello, 90146 Palermo, Italy; n.quartuccio@villasofia.it (N.Q.); s.pulizzi@villasofia.it (S.P.); s.nicolosi@villasofia.it (S.N.); d.doppido@villasofia.it (D.D.); s.ialuna@villasofia.it (S.I.); 2 Otorhinolaryngology Section, Department of Precision Medicine in Medical, Surgical and Critical Care (Me.Pre.C.C), University of Palermo, 90133 Palermo, Italy

**Keywords:** [^18^F]FDG PET, metabolic tumor volume, nasopharyngeal carcinoma, neoadjuvant chemoradiotherapy, SUVmax, total lesion glycolysis

## Abstract

**Introduction:** Nasopharyngeal carcinoma (NPC) is a distinct malignancy of the head and neck with high prevalence in endemic regions and a strong association with Epstein–Barr virus (EBV). In locally advanced stages, neoadjuvant chemotherapy (NAC) followed by chemoradiotherapy improves outcomes, but response rates vary. Identifying early predictors of NAC response is essential for guiding personalized treatment strategies. This study aims to assess whether baseline [^18^F]FDG PET/CT parameters can predict NAC response in NPC patients. **Methods:** In this retrospective study, 27 patients with histologically confirmed, locally advanced (stage III) NPC underwent baseline [^18^F]FDG PET/CT prior to NAC between 2015 and 2023. Quantitative PET parameters including SUVmax, SUVmean, metabolic tumor volume (MTV), and total lesion glycolysis (TLG) were extracted from the primary tumor. NAC response was assessed using RECIST 1.1 criteria and classified as responders (CR + PR) or non-responders (SD + PD). Group comparisons were performed using Student’s *t*-test. ROC analysis was used to identify optimal cut-off values. A *p*-value < 0.05 was considered significant. **Results:** The cohort included 20 males and 7 females (mean age: 60.8 ± 15.2 years). The predominant histotype was undifferentiated non-keratinizing carcinoma (92.6%). A total of 19 patients (70.4%) responded to NAC. Responders had significantly lower baseline SUVmax (10.9 ± 4.8 vs. 15.8 ± 4.1, *p* = 0.021), MTV (16.2 ± 12.4 vs. 27.8 ± 19.5 cm^3^, *p* = 0.045), and TLG (128.6 ± 98.2 vs. 218.7 ± 152.4, *p* = 0.038). SUVmean was also lower in responders (6.1 ± 2.1 vs. 9.3 ± 2.8), although not statistically reported. ROC analysis identified SUVmax > 12.5 and MTV > 20.0 cm^3^ as thresholds associated with poor NAC response. **Conclusions:** Baseline metabolic parameters from [^18^F]FDG PET/CT, particularly SUVmax and MTV, may assist stratification of NAC response in nasopharyngeal carcinoma. These biomarkers may facilitate pre-treatment stratification and guide more personalized therapeutic approaches. However, the limited sample size may affect the generalizability of these findings, and larger prospective studies are needed to confirm the results.

## 1. Introduction

Nasopharyngeal carcinoma (NPC) is a rare but highly distinctive malignancy within the head and neck cancer spectrum. NPC is characterized by unique epidemiological patterns, strong association with Epstein–Barr virus (EBV) infection, and pronounced geographic clustering in endemic regions including Southeast Asia, North Africa, and the Mediterranean basin [1]. In addition to its well-established link with Epstein–Barr virus infection, nasopharyngeal carcinoma has several other significant risk factors. These include tobacco use, dietary habits involving salt-preserved fish and other cured foods, hereditary factors (particularly certain HLA genetic variants), and workplace exposure to substances like wood dust and formaldehyde [2]. The incidence rates vary dramatically worldwide, with age-standardized rates exceeding 20 per 100,000 in endemic areas compared to less than 1 per 100,000 in most Western populations [3,4].

The management of locally advanced NPC has evolved significantly over the past decades [5,6]. Although radiation therapy was traditionally viewed as sufficient treatment for early-stage disease, combination treatment strategies have now become the standard approach for more advanced cases. The incorporation of simultaneous chemotherapy with radiation therapy (concurrent chemoradiotherapy) enhanced both local tumor control and patient survival rates [7]. Neoadjuvant chemotherapy has assumed a central role in the treatment of locally advanced nasopharyngeal carcinoma, allowing not only tumor volume reduction before radiation treatment but also early control of micrometastases [8,9]. This multimodal approach has demonstrated improved survival outcomes compared to radiotherapy alone; however, NAC response is not uniform among patients, making it necessary to identify reliable predictive biomarkers [10,11,12]. This heterogeneity in treatment response poses significant clinical challenges, as non-responders are exposed to unnecessary toxicity while potentially missing the window for more effective alternative treatments. Therefore, the identification of reliable predictive biomarkers for NAC response has become a critical research priority in NPC management [13].

Positron emission tomography with [^18^F]fluorodeoxyglucose ([^18^F]FDG PET) has emerged as a fundamental imaging modality in head and neck tumor evaluation, providing metabolic information that complements morphological data [14]. Previous studies in various cancer types have demonstrated the prognostic value of these PET-derived parameters, with particular emphasis on Maximum Standardized Uptake Value (SUVmax), Mean Standardized Uptake Value (SUVmean), Metabolic Tumor Volume (MTV) and Total Lesion Glycolysis (TLG) as robust predictors of treatment response and survival outcomes [12]. However, the specific predictive value of baseline PET parameters for NAC response in NPC remains incompletely characterized [15]. Increased [^18^F]FDG PET parameters, including SUVmax, MTV, and TLG indicate high tumor glucose uptake and volumetric burden, correlating with biological mechanisms that drive resistance to combined chemotherapy and radiation treatment in nasopharyngeal carcinoma [16]. Additionally, Pak and colleagues, in their systematic review and meta-analysis, underscore the strong prognostic and predictive capabilities of MTV and TLG in head and neck malignancies, further supporting their application in patient risk assessment and individualized treatment planning [15]. These biological processes establish a scientific foundation for the prognostic and predictive capabilities of PET-based parameters, supporting their promising role in personalizing treatment approaches for individual patients. A recent meta-analysis evaluated the comparative diagnostic performance of PET/CT and PET versus MRI for identifying residual disease or recurrent tumors at the primary site in nasopharyngeal carcinoma patients. The pooled data demonstrated superior sensitivity of PET-based imaging compared to MRI for tumor detection, while both modalities showed equivalent specificity in accurately identifying tumor-free patients. These results indicate that PET imaging may offer enhanced diagnostic accuracy for detecting persistent or recurrent disease, potentially improving clinical decision-making and surveillance strategies in post-treatment NPC management [2].

The primary objective of this retrospective study was to evaluate the relationship between baseline [^18^F]FDG PET quantitative parameters and histopathologic response to NAC in patients with NPC.

## 2. Materials and Methods

### 2.1. Study Design

This retrospective cohort study was conducted at the Nuclear Medicine Unit of Villa Sofia-Cervello Hospital, Palermo, Italy, in accordance with the Declaration of Helsinki and local institutional guidelines. The study protocol was approved by the institutional review board, and the requirement for informed consent was waived due to the retrospective nature of the analysis.

### 2.2. Patient Population and Inclusion Criteria

We systematically reviewed the medical records in the hospital RIS/PACS system (Elephant v.2, Agfa HealthCare, Mortsel, Belgium) of all patients diagnosed with NPC who underwent baseline [^18^F]FDG PET/CT for initial staging at our institution between January 2015 and December 2023. Inclusion criteria were: (1) histologically confirmed NPC; (2) availability of baseline [^18^F]FDG PET/CT performed within 4 weeks prior to NAC initiation; (3) completion of NAC; (4) adequate clinical follow-up data; and (5) availability of response assessment data. Exclusion criteria included: (1) prior treatment for NPC before the PET/CT scan; (2) concurrent malignancies; (3) inadequate PET image quality precluding reliable quantitative analysis; (4) diabetes mellitus with uncontrolled hyperglycemia (glucose > 200 mg/dL) at the time of PET scanning; and (5) incomplete treatment or follow-up data. All patients received platinum-based NAC regimens and radiotherapy according to institutional protocols. The treatment approach involved cisplatin (administered at 80–100 mg/m^2^ on day 1) paired with either gemcitabine (1000 mg/m^2^ given on days 1 and 8) or 5-fluorouracil (5-FU) (delivered as a continuous infusion at 1000 mg/m^2^/day over 4 consecutive days). These regimens were given in 3-week intervals for 2 to 3 treatment cycles before proceeding to concurrent chemoradiotherapy.

### 2.3. PET/CT Acquisition Protocol

All patients underwent [^18^F]FDG PET/CT imaging using a dedicated PET/CT scanner (Discovery VCT, GE Healthcare, Milwaukee, WI, USA). Patients fasted for at least 6 h. Blood glucose measurement was required to be below 150 mg/dL. Patients rested in a quiet, dimly lit room A fixed activity of [^18^F]FDG (3.7 MBq/kg) was administered intravenously. Patients, then, sat comfortably in a chair for approximately 60 min post-injection of [^18^F]FDG. The imaging acquisition started 60 ± 10 min after the radiotracer injection. CT was used for anatomical localization of PET uptake; parameters were 120 kVp, 40–120 mAs (dose modulation), 5 mm slice thickness. Then, PET acquisition started and included 3 min per bed position, ranging from the base of the skull to mid-thigh. PET images were reconstructed using ordered subset expectation maximization (OSEM) with point-spread function modeling to optimize spatial resolution.

### 2.4. Image Analysis and Quantitative PET Parameters

Image analysis was performed using dedicated software (Advantage Workstation, GE Healthcare, version 4.3, GE Healthcare, Milwaukee, WI, USA). Two nuclear medicine physicians, each with over 10 years of experience, independently reviewed all studies, and any disagreements were resolved through a consensus meeting. The primary tumor was identified and segmented using visual identification. An automatic segmentation was performed using 40% of SUVmax threshold with manual adjustment when necessary to exclude physiological uptake, in order to extract MTV. The 40% SUVmax threshold was selected based on its established validation in head and neck cancer literature and its demonstrated correlation with clinical outcomes [17,18,19]. The following parameters were automatically calculated for each segmented tumor: SUVmax, SUVmean, MTV, TLG. Standardized uptake values (SUV) were normalized by patient body weight (SUVbw), as this represents the standard approach in our institution.

### 2.5. NAC Response Assessment

Response to NAC was evaluated based on morphological changes observed on post-treatment CT scans using RECIST 1.1 criteria. According to these morphologic criteria, patients were classified as responders if they achieved complete response (CR) or partial response (PR), and as non-responders if they showed stable disease (SD) or progressive disease (PD). The evaluation was based exclusively on anatomical changes in tumor size following NAC, as measured on CT images. Baseline PET/CT parameters were used solely as predictive biomarkers of subsequent morphological response and were not employed for direct response classification. For statistical analyses, patients were dichotomized into responders (CR + PR) and non-responders (SD + PD).

### 2.6. Statistical Analysis

Statistical analyses were performed using SPSS version 28.0 (IBM Corp., Armonk, NY, USA). Continuous variables were presented as mean ± standard deviation or median (range) as appropriate. Categorical variables are expressed as frequencies and percentages. Comparisons between groups were performed using Student’s *t*-test for continuous variables and chi-square test for categorical variables. ROC curve analysis was performed to determine optimal cut-off values for PET parameters. A *p*-value < 0.05 was considered statistically significant. All tests were two-tailed.

## 3. Results

### 3.1. Patient Characteristics

The final study cohort comprised 27 patients (20 males and 7 females) with a mean age of 60.8 years. The undifferentiated non-keratinizing subtype was the most frequent histotype, and the majority of patients had T3/N2 stage disease at diagnosis. All patients were classified as stage III according to TNM 8th edition [20]. The median Ki-67 proliferation index was 50% (range: 20–80%). EBER (Epstein–Barr Virus Early RNA) was positive in 7 patients (25.9%) and negative in 20 patients (74.1%). The demographic and clinical characteristics of the study population are summarized in Table 1.

### 3.2. Baseline PET/CT Parameters

Baseline PET/CT parameters are detailed in Table 2. The distribution of SUVmax, SUVmean, MTV, and TLG showed considerable variability across the cohort, with SUVmax averaging 12.4 ± 5.2.

### 3.3. NAC Response

A total of 19 patients (70.4%) demonstrated a response to neoadjuvant chemotherapy, whereas 8 patients (29.6%) were classified as non-responders. Among the responders, 12 patients (63.2%) achieved a partial response, while 7 patients (36.8%) achieved a complete response.

### 3.4. Correlation Between PET Parameters and NAC Response

A statistically significant correlation was observed between baseline PET parameters and NAC response (Table 3). Responders demonstrated significantly lower baseline values in SUVmax (10.9 ± 4.8 vs. 15.8 ± 4.1, *p* = 0.021), MTV (16.2 ± 12.4 vs. 27.8 ± 19.5, *p* = 0.045), and TLG (128.6 ± 98.2 vs. 218.7 ± 152.4, *p* = 0.038) compared to non-responders. A trend toward lower SUVmean values was also observed among responders (6.1 ± 2.1 vs. 9.3 ± 2.8), although this difference did not reach statistical significance.

**Figure 1:**
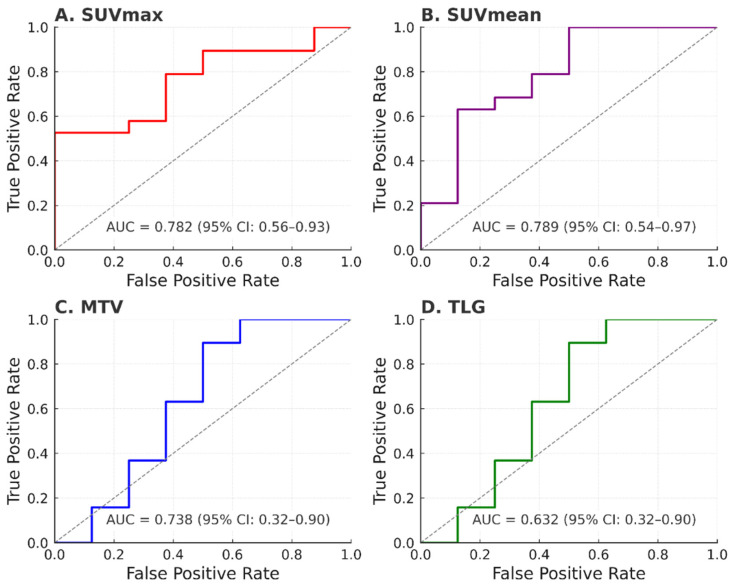
Roc Curves of (**A**) SUVmax, (**B**) SUVmean, (**C**) MTV and (**D**) TLG for predicting NAC response in patients with NPC. The cut-off values were determined using ROC analysis to optimize the balance between sensitivity and specificity for predicting response to NAC.

Receiver operating characteristic (ROC) curve analysis confirmed the predictive value of baseline PET parameters. SUVmax showed the highest discriminative ability (AUC = 0.782), followed by MTV (AUC = 0.738), SUVmean (AUC = 0.724), and TLG (AUC = 0.632). Optimal cut-off values and corresponding sensitivity/specificity measures are reported in Table 4 and Figure 1.

## 4. Discussion

The main advantage of PET-CT scans is their capacity to identify diseases at an early stage, a factor that can be pivotal for timely and effective treatment. By enabling early diagnosis, they support the development of more targeted therapeutic strategies and improve patient outcomes. Furthermore, PET-CT plays a crucial role in cancer staging and in monitoring treatment response, thanks to its ability to reveal metabolic changes that may remain undetectable with conventional imaging methods [21].

This study evaluated the predictive role of baseline [^18^F]FDG PET/CT metabolic parameters in NAC response in patients with NPC. Among the four parameters analyzed, SUVmax, metabolic tumor volume (MTV), and total lesion glycolysis (TLG) demonstrated significant correlations with treatment response, whereas SUVmean did not achieve statistical significance. The observed association between higher baseline SUVmax and MTV values and poor response to neoadjuvant chemotherapy (NAC) in nasopharyngeal carcinoma (NPC) patients may be explained by complex underlying biological mechanisms [22]. While elevated SUVmax typically reflects increased tumor glucose metabolism and aggressiveness, it does not universally indicate chemoresistance, as many aggressive tumors with high metabolic activity respond well to chemotherapy. The heterogeneous nature of tumors means that in some cases high SUV and MTV may be linked to factors such as tumor hypoxia, cellular heterogeneity, and activation of survival pathways, which contribute to resistance to chemoradiotherapy [22,23].

Our findings are consistent with previous studies in head and neck cancers [15,16,24,25]. Volumetric parameters such as MTV and TLG have repeatedly shown stronger prognostic power than single-voxel metrics like SUVmax alone. For instance, Chan et al. demonstrated that high MTV predicted poorer survival outcomes in NPC treated with concurrent chemoradiotherapy, supporting volumetric assessment as a reflection of tumor burden and aggressiveness [24]. Similar observations have been reported in oral cavity and oropharyngeal carcinomas, where MTV outperformed SUV-derived indices for treatment outcome prediction [15,16]. In our study, SUVmax maintained the highest AUC among parameters, suggesting that peak metabolic activity is a robust indicator of biological aggressiveness and chemoresistance, aligning with prior studies associating high SUVmax with poor response and prognosis [25]. Conversely, SUVmean did not show significant association with NAC response. This may be explained by its averaging nature, which can obscure focal areas of intense glycolytic activity particularly relevant in highly heterogeneous tumors such as NPC. Because nasopharyngeal tumors frequently display infiltrative and asymmetric uptake within anatomically complex regions, SUVmean might underestimate the true metabolic heterogeneity and aggressiveness, diminishing its predictive sensitivity [26]. This supports the emerging consensus that volumetric and peak activity PET parameters possess superior sensitivity for therapeutic response prediction.

The establishment of clinical thresholds (SUVmax > 12.5, MTV > 20 cm^3^ in our study) has direct translational implications. Implementing these cut-offs in clinical pathways may facilitate early stratification of patients into high- and low-likelihood responders to NAC. Patients exceeding these values might benefit from intensified or alternative treatment regimens such as novel systemic therapies, earlier radiotherapy, or incorporation in clinical trials evaluating targeted agents or immunotherapies. This stratification could optimize resource allocation, minimize exposure to ineffective treatment toxicity, and personalize management [27]. Nonetheless, these thresholds require prospective validation in larger, multicenter cohorts before routine clinical adoption. When evaluating PET metabolic parameters as predictive tools for NPC, it is crucial to consider how they compare and relate to other well-established biomarkers like plasma EBV DNA levels. EBV DNA serves as an effective prognostic indicator and treatment response monitor by reflecting overall tumor load throughout the body, but it cannot capture variations in tumor characteristics within different regions of the same tumor [28]. In contrast, volumetric PET parameters measure overall metabolic activity, reveal how the disease is distributed spatially and indicate local tumor aggressiveness [29]. This makes PET imaging potentially valuable as a complement to blood-based biomarkers rather than a replacement. Moving forward, research should focus on combining metabolic imaging findings with blood test results in comprehensive predictive models, which could lead to more precise patient risk assessment and treatment planning.

Several limitations should be acknowledged in interpreting these results. The retrospective nature of the study and relatively small sample size limit the generalizability of findings. The heterogeneity in chemotherapy regimens, although reflecting real-world clinical practice, may have influenced response rates. Additionally, the threshold methodology for MTV calculation (40% of SUVmax) was fixed rather than optimized for this specific population. The study cohort predominantly consisted of patients with undifferentiated non-keratinizing carcinoma (92.6%), while only a small minority (7.4%) had squamous cell carcinoma. Due to the very limited number of cases with squamous cell carcinoma (n = 2), a subgroup analysis according to histological subtype was not feasible. Consequently, the potential influence of histological heterogeneity on the study results cannot be fully assessed. The limited number of patients in the study prevented the use of multivariate logistic regression to control for potential confounding variables including age, sex, EBV status, and Ki-67 proliferation index. Larger-scale investigations involving more extensive patient populations are needed to enable comprehensive multivariate statistical modeling, which would provide a more definitive assessment of whether baseline PET/CT metrics can independently predict response to neoadjuvant chemotherapy in nasopharyngeal carcinoma patients. The relatively short follow-up period limits our ability to assess long-term survival outcomes and their correlation with PET parameters. Future prospective studies with larger cohorts and standardized treatment protocols are needed to validate these findings. Future research will focus on using artificial intelligence (AI) and radiomics to analyze PET/CT imaging data. Radiomics enables researchers to extract numerous quantitative measurements from medical images, capturing the variability in radiotracer distribution at a level of detail that traditional metrics like SUVmax or MTV cannot achieve [30]. AI algorithms could be developed to recognize intricate patterns in these radiomic features that correlate with patients who do not respond to neoadjuvant chemotherapy. Once these AI models are properly tested and validated, they could be incorporated into clinical software systems to automatically generate individualized risk assessments for each patient, a concept that has already been explored in other cancer specialties. This approach would provide clinicians with personalized, computer-generated predictions to guide treatment decisions [31]. The integration of artificial intelligence (AI) in diagnostic imaging is rapidly evolving and holds significant promise for NPC management. As highlighted by Cergan et al. [32], the future of diagnostic and interventional imaging is poised to be transformed by AI-powered tools that enhance workflow efficiency in increasingly burdened radiology departments. In the context of NPC, such AI systems could facilitate rapid lesion detection, automated tumor contouring, structured reporting, and treatment response monitoring. Notably, these capabilities could support individualized treatment planning and follow-up strategies, especially in settings with high patient volumes. Future research efforts should therefore focus on validating AI-based models in NPC—especially in head and neck oncology—addressing challenges like imaging standardization, external validation, and integration into clinical decision support systems.

## 5. Conclusions

This study highlights the potential role of baseline ^18^F-FDG PET/CT metabolic parameters for assisting in the stratification of NAC response in NPC. Patients with SUVmax above 12.5 and MTV greater than 20 cm^3^ exhibited significantly lower response rates, indicating a potential need for alternative therapeutic strategies in this subgroup. Further prospective studies are needed to validate these findings and to investigate the added value of combining PET parameters with other biomarkers to enhance patient selection for NAC.

## Figures and Tables

**Table 1:** Patient demographics and clinical characteristics.

Characteristic	N (%) or Mean ± SD
Age (years)	60.8 ± 15.2
Sex—Male	20 (74.1%)
Sex—Female	7 (25.9%)
Histotype—Undifferentiated non-keratinizing	25 (92.6%)
Histotype—Squamous cell carcinoma	2 (7.4%)
T Stage—T3	20 (74.1%)
T Stage—T4	7 (25.9%)
N Stage—N1	4 (14.8%)
N Stage—N2	21 (77.8%)
N Stage—N3	2 (7.4%)
AJCC Stage—III	27 (100%)
EBER Positive	7 (25.9%)
EBER Negative	20 (74.1%)
Ki-67 Proliferation Index	Median: 50% (range 20–80%)

**Table 2:** Baseline PET Parameters.

Parameter	Mean ± SD	Range
SUVmax	12.4 ± 5.2	4.1–33.0
SUVmean	6.8 ± 2.8	3.6–12.9
MTV (cm^3^)	19.8 ± 16.2	5.7–63.2
TLG (g/mL × cm^3^)	156.4 ± 128.7	22.2–423.9

**Table 3:** Comparison between responders and non-responders; ns.

Parameter	Responders (n = 19)	Non-Responders (n = 8)	*p*-Value
SUVmax	10.9 ± 4.8	15.8 ± 4.1	0.021
SUVmean	6.1 ± 2.1	9.3 ± 2.8	ns
MTV (cm^3^)	16.2 ± 12.4	27.8 ± 19.5	0.045
TLG (g/mL × cm^3^)	128.6 ± 98.2	218.7 ± 152.4	0.038

**Table 4:** ROC analysis of baseline PET parameters for prediction of NAC response.

Parameter	Cut-Off	Sensitivity (%)	Specificity (%)	AUC (95% CI)
SUVmax	12.5	75.0	78.9	0.782 (0.598–0.966)
SUVmean	7.8	62.5	78.9	0.724 (0.521–0.928)
MTV (cm^3^)	20.0	62.5	84.2	0.738 (0.539–0.936)
TLG	145.0	75.0	63.2	0.632 (0.405–0.860)

## Data Availability

The data presented in this study are available on request from the corresponding author.

## Abbreviations

AI	Artificial Intelligence
AUC	Area Under the Curve
CCRT	Concurrent Chemoradiotherapy
CR	Complete Response
CT	Computed Tomography
EBER	Epstein–Barr Virus Early RNA
EBV	Epstein–Barr Virus
FDG	Fluorodeoxyglucose
IC	Induction Chemotherapy
MRI	Magnetic Resonance Imaging
MTV	Metabolic Tumor Volume
NAC	Neoadjuvant Chemotherapy
NPC	Nasopharyngeal Carcinoma
OSEM	Ordered Subset Expectation Maximization
PD	Progressive Disease
PET/CT	Positron Emission Tomography/Computed Tomography
PR	Partial Response
RECIST	Response Evaluation Criteria in Solid Tumors
ROC	Receiver Operating Characteristic
SD	Stable Disease
SUVmax	Maximum Standardized Uptake Value
SUVmean	Mean Standardized Uptake Value
TLG	Total Lesion Glycolysis
